# Inorganic Nitrogen Enhances the Drought Tolerance of Evergreen Broad-Leaved Tree Species in the Short-Term, but May Aggravate Their Water Shortage in the Mid-Term

**DOI:** 10.3389/fpls.2022.875293

**Published:** 2022-04-25

**Authors:** Fangyan Liu, Yuheng Zhou, Shike Zhang, Nan Liu

**Affiliations:** ^1^Chinese Academy of Sciences Engineering Laboratory for Vegetation Ecosystem Restoration on Islands and Coastal Zones, South China Botanical Garden, Chinese Academy of Sciences, Guangzhou, China; ^2^College of Life Sciences, Gannan Normal University, Ganzhou, China; ^3^Southern Marine Science and Engineering Guangdong Laboratory, Guangzhou, China; ^4^University of Chinese Academy of Sciences, Beijing, China

**Keywords:** functional traits, photosynthesis, resistance, climate change, stress, interaction

## Abstract

With global climate change, atmospheric nitrogen (N) deposition and drought have been well documented to cause substantial challenges for tropical and subtropical evergreen broad-leaved forests. Here, we conducted an experiment that measured the physiological responses of the seedlings of three dominant tree species (*Tabebuia chrysantha*, *Elaeocarpus sylvestris*, and *Bischofia javanica*) of the evergreen broad-leaved forests in South China under control (CT), drought stress (D), N addition (N), and drought stress plus N addition (N+D). We found that N addition significantly decreased malondialdehyde (MDA) content, abscisic acid (ABA) content, total antioxidant capacity (T-AOC), but significantly increased the content of proline (PRO), and the activities of ribulose-1, 5-bisphosphate carboxylase/oxygenase (Rubisco), nitrate reductase (NR), nitrite reductase (NiR), and glutamine synthetase (GS) in the three species under D. Meanwhile, we also found that under drought conditions, N addition promoted the leaf transpiration rate (E), stomatal conductance (g_*s*_), and light-saturated net photosynthetic rate (A_*max*_) of the three species. These results indicate that N addition can enhance the drought tolerance of the three species by osmotic adjustment and protecting the photosystem. However, the enhancement in A_*max*_ and E will cause plants to face more severe drought conditions, especially *B. javanica* (large tree species). This study helps to explain why the evergreen broad-leaved forests in South China are gradually degrading to shrublands in recent decades.

## Introduction

A large amount of evidence shows that climate change and human disturbance have emerged as the most serious challenges faced by tropical and subtropical evergreen broad-leaved forests, such as extreme precipitation, severe drought, fire, and storm (Davidson et al., 2012). There is a growing consensus that increased climate change affects the material cycle, energy flow, and community structure of forest ecosystems, which further affects the service function of tropical and subtropical forest ecosystems (Zhou et al., 2014; Millar and Stephenson, 2015). Although the total amount of annual precipitation in South China has increased over the past few decades, the dry season has become drier while the wet season has become wetter, which has led to the increase of severe drought events (Zhou et al., 2011; Chou et al., 2013). Severe drought has been well documented to increase the mortality of trees and thus threaten the carbon sink of terrestrial ecosystems (Phillips et al., 2009; Allen et al., 2010; Michaelian et al., 2011). Severe drought can cause osmotic stress to plants, which leads to the destruction of cell structure and consequently affects plant growth and even results in the death of plants (Bartels and Sunkar, 2005). In response to water deficiency conditions, plants normally close their stomata to moderate dehydration and avoid xylem cavitation (Martin-Stpaul et al., 2017). However, on the other hand, the closure of stomata will lead to the rapid weakening of photosynthetic carbon assimilation, weaken the ability of the canopy to dissipate heat through transpiration, and cause higher photodamage to leaves (Choat et al., 2018). Then, plants will increase photorespiration, antioxidant enzyme activity, and antioxidant production to reduce the damage (Pankovic et al., 1999; Makino, 2011). Meanwhile, plants secrete osmolytes, such as proline and soluble carbohydrates, to maintain cell water potential (Ashraf and Foolad, 2007; Zhong et al., 2019). Many studies have indicated that the adaptability and resistance of plants to drought are not unlimited (Schwalm et al., 2017). Frequent drought induces the mortality of trees and leads to community degradation (Schwalm et al., 2017; Choat et al., 2018).

Since the industrial revolution, industrial emissions and fertilizer use have been increasing, resulting in increased nitrogen (N) emissions, transport, and deposition (Liang et al., 2020). With the rapid industrial and social development, the N deposition rate in South China has become one of the highest in the world (Bobbink et al., 2010). In 2010, the natural rate of N deposition in the evergreen broad-leaved forests in South China reached 24.4 kg N ha^–1^year^–1^ and was predicted to exceed 50 kg N ha^–1^year^–1^ by 2030 (Table 1) (Bobbink et al., 2010). Excessive levels of N deposition have been well documented to cause a series of detrimental effects, for example, affecting plant growth (Mo et al., 2008) and productivity (Yu et al., 2016), leading to soil acidification (Lu et al., 2010), changes in N cycle (Liu et al., 2011), and forest degradation (Magill et al., 2004). Many studies have indicated that N deposition can enhance photosynthesis by promoting Rubisco enzyme content, increasing the demand for carbon dioxide (CO_2)_ and heat dissipation, and inducing stomatal opening and transpiration (Lu et al., 2010; Schulte-Uebbing and de Vries, 2018; Liang et al., 2020). The difference in soil N situation will lead to different effects of N on the ecosystem. In N-insufficient ecosystems, N deposition mostly brings beneficial effects on plant growth (Guerrieri et al., 2011), while in N-rich ecosystems, long-term N deposition will reduce soil pH, thus reducing the availability of calcium (Ca^2+^), magnesium (Mg^2+^), and other basic cations, and even precipitate aluminum ions which will produce aluminum toxicity on plant roots and ultimately inhibit plant growth (Lu et al., 2010; Lu et al., 2018). Under different water conditions, the influence of N on the ecosystem was also found to be discrepant. In dry ecosystems, N deposition commonly weakens mineralization, reduces plant drought-related diseases, and makes water the most limiting factor for plant growth, while in wet ecosystems, N deposition normally enhances mineralization and increases plant moisture-related diseases (Greaver et al., 2016).

**TABLE 1:** The change of nitrogen deposition rate in South China from 1990 to 2010 and the predicted value of nitrogen deposition rate in 2030 (Bobbink et al., 2010).

	1990–2000	2010	2030 (Predictive value)
N deposition rate in south China	19.7 kg N ha^–1^year^–1^	24.4 kg N ha^–1^year^–1^	>50 kg N ha^–1^year^–1^

Nowadays, there is research on the effects of severe drought, N deposition, and their co-effects on plant growth in different ecosystems (Allen et al., 2010; Zhu et al., 2013; Li Y. et al., 2020; Liang et al., 2020). Existing studies have shown that N deposition can enhance plant tolerance to drought by the promotion of antioxidant enzyme activity, osmolytes, and photosynthesis (Cerezini et al., 2017; Sigala et al., 2020). But this mitigation was found to be unsustainable (Greaver et al., 2016; Liang et al., 2020; Wang et al., 2021). The main reason may be the positive effect of N supply on plant water consumption (Abid et al., 2016; Lu et al., 2018). However, in the current and previous studies, there is still a lack of understanding on plant N metabolism under drought-N complex conditions (Planchet et al., 2011; Cao et al., 2018). Moreover, there has been very little research on the adaptation strategies of different subtropical forest plant species to N deposition under water shortage (Abid et al., 2016; Shi et al., 2017; Zhong et al., 2019; Iqbal et al., 2020).

In this study, the seedlings of three dominant tree species (*Tabebuia chrysantha*, *Elaeocarpus sylvestris*, and *Bischofia javanica*) in an evergreen broad-leaved forest (mainly N-rich soils) in South China were selected under drought and/or N addition. N deposition is a continuous and long-term phenomenon, while the climate in South China has a periodic change of alternating dry and wet seasons (Zhou et al., 2014). Under the condition of continuous drought in the dry season, it is not clear how N deposition will affect the growth and physiological status of forest plants. Thus, in this study, we attempted to figure out (1) What are the physiological effects of drought and N addition on N metabolism and physiological characteristics of the dominant subtropical forest plants; (2) How does N addition affect the acclimation of subtropical forest plants in response to extreme drought. Results from this study may help in explaining the dynamics of subtropical evergreen broad-leaved forest under the background of global change in terms of N deposition and extreme drought.

## Materials and Methods

### Plant Materials

The tree species (*T. chrysantha*, *E. sylvestris*, and *B. javanica*) selected for this research are the three dominant tree species distributed in subtropical evergreen broad-leaved forests. *T. chrysantha* is a deciduous small tree, 4–5 m high, adapted to high temperatures, and distributed in Mexico, Central America, South America, and tropical and subtropical regions of China. *E. sylvestris* is a small shade-tolerant tree with a height of about 10 m, suitable for growing in the valley dense forest environment with moist and deep soil layer, and is often a middle-level tree in dense forests, distributed in subtropical regions of China, growing in the evergreen forest with an altitude of 350–2,000 m, and also distributed in Vietnam, Laos, and Thailand. *B. javanica* is large evergreen or semi-evergreen tree, up to 40 m high, often grows in mountainous humid valley forests below 800 m above sea level, and the young trees are slightly shade-tolerant and adapted to humidity. *B. javanica* is one of the main tree species in the upper layer of tropical and subtropical evergreen seasonal rain forests and are distributed in Australia, Polynesia, Cambodia, Myanmar, Thailand, Laos, Vietnam, Malaysia, Indonesia, Philippines, Japan, India, and China.

### Treatments

In this experiment, all seedlings were 20–40 cm high. All the seedlings were planted in pots (the bottom diameter is 14 cm, the upper diameter is 16 cm, and the height is 18 cm) and fully maintained in greenhouse conditions. During this period, we did not transplant seedlings, cut branches, or hurt their roots. In the first week, we simply watered and observed the seedlings we bought and eliminated the seedlings whose growth was significantly weakened or did not adapt to the greenhouse environment. The remaining seedlings were observed for 3 days before the formal experiment.

Seedlings of each species were divided into four groups: control group (CT), N addition plus drought group (N+D), N addition group (N), and drought group (D). Before the formal experiment, we carried out three rounds of pre-experiments according to the same greenhouse conditions, pot conditions, seedling types, and treatment methods. It was found that a large number of leaves withered and fell off in the three tested species from day 6 to 7 of D treatment. Based on this, we set the period of the formal experiment as 7 days to track and observe the whole process of the response of these tree species to different treatments.

A total of 25 pots of seedlings of each treatment were placed on the greenhouse benches for a 7-days experiment (day 0 to 7). On day 0 at dusk, the seedlings of the CT group and N group were watered until the water flowed out from the bottom. The CT seedlings were watered in the same way every other day until day 7 (not watered on day 1). The N seedlings were also watered every other day at dusk until day 7 (not watered on day 1), and from day 1, N solution (10 ml of 20 mM NH_4_NO_3_) was applied to each pot of N group every odd-numbered day at nightfall until day 7. The D seedlings were not watered from day 0 to 7. The N+D seedlings were not watered as the D group and applied N the same way with the N group every odd-numbered day until day 7. The experiment schedule is shown in Table 2.

**TABLE 2:** Timing of water and nitrogen application for the four groups of the treatments in this experiment.

Day	Treatment			
	CT	N+D	D	N
0	Water	-	-	Water
1	-	NH_4_NO_3_	-	NH_4_NO_3_
2	Water	-	-	Water
3	-	NH_4_NO_3_	-	NH_4_NO_3_
4	Water	-	-	Water
5	-	NH_4_NO_3_	-	NH_4_NO_3_
6	Water	-	-	Water
7	-	NH_4_NO_3_	-	NH_4_NO_3_

*CT, control group; N+D, drought plus nitrogen addition group; D, drought group; N, nitrogen addition group.*

The N addition rate in this experiment equaled 50 kg N ha^–1^year^–1^. The amount of N application converted was 10 ml of 20 mM NH_4_NO_3_ every time in this experiment, and the effect of NH_4_NO_3_ solution on soil water potential under drought conditions is negligible in this study (Guo et al., 2010; Liu et al., 2016).

The leaf samples selected in this experiment were located on the seedling branches, from the third to eigth leaves from the tip. The leaf samples were taken from three different seedlings of each species once a day, then wrapped with aluminum foil, soaked in liquid N, and stored in a refrigerator set at −80°C.

### Measurements on Leaf Gas Exchange

The leaf transpiration rate (E), stomatal conductance (g_*s*_), light-saturated net photosynthetic rate (A_*max*_), and intercellular CO_2_ concentration (C_*i*_) were measured by a portable photosynthesis system (LI-6800, Li-Cor, Inc., Lincoln, SEC, United States) with room temperature, 400 μmol mol^–1^ of CO_2_, and 1,500 μmol m^–2^ s^–1^ photon flux density (Liu et al., 2018; Liu et al., 2021). Measurements were made from three leaves on different seedlings for each treatment and species between 9:00 and 11:00 every day.

Instantaneous carboxylation efficiency of CO_2_ assimilation (CE_*i*_), intrinsic water-use efficiency (WUE_*i*_), and instantaneous water-use efficiency (WUE_*n*_) were respectively calculated by CE_*i*_ = A_*max*_/C_*i*_, WUE_*i*_ = A_*max*_/g_*s*_, and WUE_*n*_ = A_*max*_/E (Field et al., 1982; Field et al., 1983).

### Measurements on Ribulose-1, 5-Bisphosphate Carboxylase/Oxygenase (Rubisco) Activitiy

Rubisco activity was measured from the leaf samples stored in the refrigerator. We ground and homogenized a 0.1 g leaf sample in an extraction buffer that contains 5 mm MgCl_2_, 1 mm Ethylene Diamine Tetraacetic Acid (EDTA), 5 mm dithiothreitol (DTT), 100 mm Bicine (pH 7.8), and 0.002% BSA (Bovine albumin) (w/v). In total, 5 μl of the extract are then mixed with 745 μl assay buffer that consist of 15 mm MgCl_2_, 9.2 mm NaHCO_3_, 18.5 mm NaCl, 1 mM EDTA, 9.2 mm DTT, 0.6 mM Ribulose-1,5-disphosphate (RuBP), 50 mM Bicine pH 8.0, 0.4 mm Nicotinamide adenine dinucleotide (NADH), 0.5 mm ATP, 1.3 U of phosphocreatine kinase, 4.6 mM phosphocreatine, 47 U of glyceraldehyde 3-phosphate dehydrogenase (GAPD), and 47 U of phosphoglycerate kinase (PGK). The reading when the absorbance was monitored at 340 nm for 2 min was recorded (Sharkey et al., 1991). One leaf per treatment per species was measured between 9:00 and 11:00 every day. The middle of the blade is selected as the measuring position, avoiding the position of the leaf vein.

### Measurements on Nitrogen Assimilation Enzyme Activity

Nitrate Reductase (NR) was measured from the leaf samples stored in the refrigerator. Homogenized 0.1 g leaf sample into 1 ml of phosphate buffer (pH 8.7) consisting of 10 mm cysteine and 1 mM EDTA (Ethylene Diamine Tetraacetic Acid). Then, 10 min were used to centrifuge the homogenate at 8,000 *g* and mixed the supernatant 10 mm NADH with 20 mM KNO_3_. After 30 min of waiting, sulfanilamide and N (1-naphthyl) ethylene diamine dihydrochloride were added to terminate the reaction. Finally, the absorbance value at 540 nm was recorded with a spectrophotometer (Kaiser and Brendle-Behnisch, 1991; Liu et al., 2018). One leaf per treatment per species was measured between 9:00 and 11:00 every day. The middle of the blade is selected as the measuring position, avoiding the position of the leaf vein.

Nitrite reductase (NiR) and glutamine synthetase (GS) were measured from the leaf samples stored in the refrigerator. Homogenized 0.1 g leaf sample and 1 ml of Tris-HCl (pH 7.8) consisting of 0.1% Triton X-100, 15% glycerol, 1 mm EDTA, and 14 mm 2-mercaptoethanol. Then, 10 min was taken to centrifuge the homogenate at 8,000 *g*. NiR activity could be obtained by measuring the reduction of NO_2_^–^ at 520 nm (Lillo, 1984). Acidified ferric chloride was added to the homogenate, and GS activity could be obtained at 540 nm by measuring the formation of glutamyl hydroxamate in the supernatant (Kaiser and Lewis, 1984). One leaf per treatment per species was measured between 9:00 and 11:00 every day. The middle of the blade is selected as the measuring position, avoiding the position of the leaf vein.

### Measurements on Abscisic Acid Concentration

The abscisic acid (ABA) concentration of the leaf samples stored in the refrigerator was measured by high-performance liquid chromatography (HPLC) (Murakami-Mizukami et al., 1991). The 0.5 g leaf sample was mixed and homogenized with 80% cold methanol at first. Then the homogenate was filtered. The residue was resuspended in the methanol, then incubated in the dark for 1 h, and filtered again. The two filtrates were blended, and mixed with PVPP after being dried by evaporation with N. The suspension was filtered, and its pH was adjusted to 3.0, and then the three same volumes of petroleum ether were used to wash for three consecutive times. Extracted the ABA from the water layer by diethyl ether dried by evaporation with N three consecutive times. Under the condition of HPLC (RIGOL L3000, kromasil C18 column, 4.6 mm × 250 mm), an ultraviolet (UV) detector was used. The flow rate was set at 1.0 ml/min, the wavelength was 254 nm, and the column temperature was 40°C. One leaf per treatment per species was measured daily. The middle of the blade is selected as the measuring position, avoiding the position of the leaf vein.

### Measurements of Proline Concentration

Proline (PRO) was extracted from the leaf samples stored in the refrigerator by sulfosalicylic acid. A total of 0.5 g leaf sample was mixed into 5 ml 3% sulfosalicylic acid aqueous solution. Then anhydride and glacial acetic acid were added. Then the extract was filtered and heated in a water bath for 1 h at 100°C. The absorption value at 520 nm was read and recorded after extraction with toluene of the mixture (Bates et al., 1973). One leaf per treatment per species was measured between 9:00 and 11:00 every day. The middle of the blade is selected as the measuring position, avoiding the position of the leaf vein.

### Measurements on Malondialdehyde Concentration

According to the thiobarbituric acid method (Cakmak and Horst, 1991), a 0.2 g leaf sample from the refrigerator was blended into 5 ml of 10% TCA. Then, the mixture was centrifuged at 10,000× *g* for 20 min and then added 2 ml supernatant into 2 ml of 6% 2-thiobarbituric acid. The absorption values were measured at 450, 532, and 600 nm with a spectrophotometer. Malondialdehyde (MDA) concentration was obtained by C = 6.45 (A_532_ − A_600_) − 0.56A_450_. One leaf per treatment per species was measured between 9:00 and 11:00 every day. The middle of the blade is selected as the measuring position, avoiding the position of the leaf vein.

### Measurements on Total Antioxidant Capacity

The leaf samples were taken from the refrigerator. 0.5 g leaf sample was ground and precipitated in 5 ml distilled water, and then 3 ml of the mixture was centrifuged at 10,000× *g* under 4°C for 10 min. Under acidic conditions, Fe^3+^-tripyridine triacridine (TPTZ) could be reduced to Fe^2+^-TPTZ in the supernatant and displayed in blue. The total antioxidant capacity (T-AOC) could be obtained by measuring the absorption value of the production of blue substances at 593 nm (Benzie and Strain, 1996). One leaf per treatment per species was measured between 9:00 and 11:00 every day. The middle of the blade is selected as the measuring position, avoiding the position of the leaf vein.

### Data Analysis

In this experiment, for MDA, the Rubisco, ABA, PRO, NR, NiR, GS, and T-AOC of each species in each group and the data measured by seven leaves in 7 days during the experiment were analyzed as seven repetitions. For the gas exchange data, three leaves measured every day are taken as three repetitions for each species in each group. All statistical analysis was generated with R version 3.6.3^1^ and SPSS 23.0^2^ (IBM, NY, United States). Two-way repeated-measures ANOVA was applied to compare the effects of treatment, species, and the interaction between them in each combination. Fisher’s least significant difference (LSD) tests were applied to compare the effects on different treatments in each species.

## Results

### Physiological Indices

**FIGURE 1:**
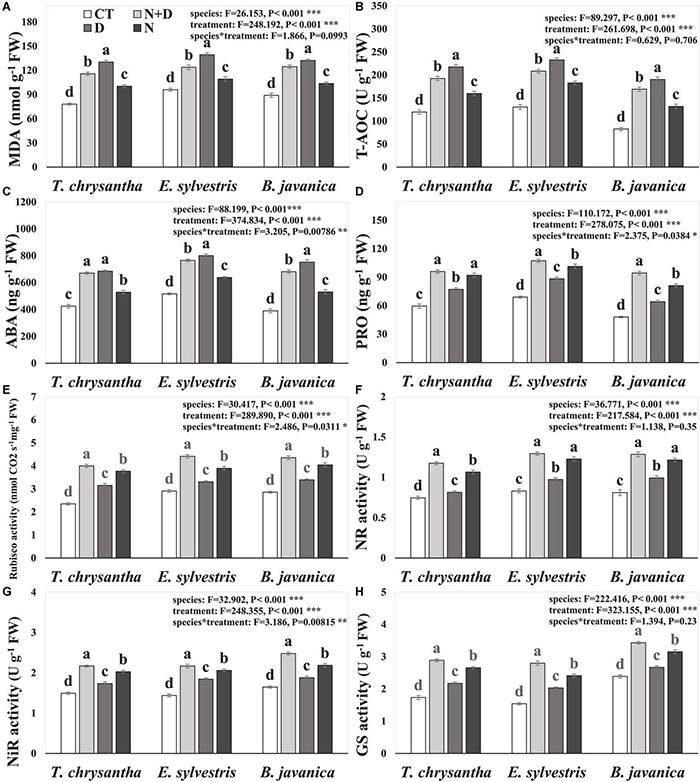
Malondialdehyde (MDA) concentration **(A)**, total antioxidant capacity (T-AOC) **(B)**, abscisic acid (ABA) concentration **(C)**, proline (PRO) concentration **(D)**, ribulose-1, 5-bisphosphate carboxylase/oxygenase (Rubisco) activity **(E)**, nitrate reductase (NR) activity **(F)**, nitrite reductase (NiR) activity **(G)**, and glutamine synthetase (GS) activity **(H)** of *Tabebuia chrysantha*, *Elaeocarpus sylvestris*, and *Bischofia javanica* in response to control (CT), nitrogen addition plus drought(N+D), drought (D), and nitrogen addition (N) treatments. Different letters indicated the significant difference by Fisher’s least significant difference (LSD) test (*P* < 0.05). Error bars were represented standard error (SE).

Compared to the CTs, the MDA concentration, T-AOC, and ABA concentration of *T. chrysantha, E. sylvestris*, and *B. javanica* increased significantly in response to N+D, D, and N treatments, and except for the ABA concentration of *T. chrysantha*, the values under D were significantly higher than under N+D (Figures 1A–C). The PRO concentration of *T. chrysantha, E. sylvestris.* and *B. javanica* significantly increased under N+D, D, and N treatments (Figure 1D). The Rubisco, NR, NiR, and GS activities of *T. chrysantha, E. sylvestris*, and *B. javanica* were obviously elevated under D and increased significantly under N+D and N treatments, moreover, the values under N+D were the highest (Figures 1E–H).

### Linear Regression

**FIGURE 2:**
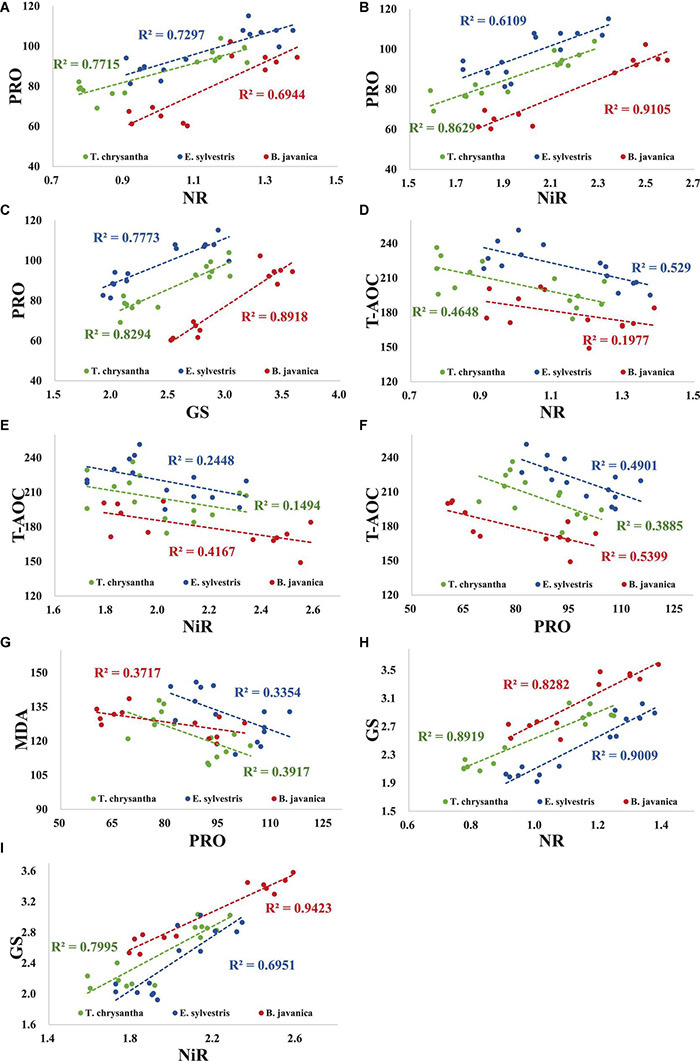
Correlation among physiological indices of *Tabebuia chrysantha*, *Elaeocarpus sylvestris*, and *Bischofia javanica* under nitrogen addition plus drought (N+D) and drought (D) treatments. PRO concentration vs. NR activity **(A)**, PRO concentration vs. NiR activity **(B)**, PRO concentration vs. GS activity **(C)**, T-AOC vs. NR activity **(D)**, T-AOC vs. NiR activity **(E)**, T-AOC vs. PRO concentration **(F)**, MDA concentration vs. PRO concentration **(G)**, GS activity vs. NR activity **(H)**, GS activity vs. NiR activity **(I)**.

Under N+D and D treatments, there was a significant positive correlation between the PRO concentration and the activities of NR, NiR, and GS in three tree species (Figures 2A–C). T-AOC was apparently negatively correlated with NR activity in *T. chrysantha* and *E. sylvestris*, but not in *B. javanica* (Figure 2D). The T-AOC was significantly negatively correlated with NiR activity in *B. javanica*, but not in *T. chrysantha* and *E. sylvestris* (Figure 2E). The T-AOC of the three species were significantly negatively correlated with PRO concentration (Figure 2F). The MDA concentration was significantly negatively correlated with PRO concentration in each species (Figure 2G). In addition, there was a significant negative correlation between GS activity and NR or between GS activity and NIR activity in the three plants (Figures 2H,I).

### Gas Exchange Indices

**FIGURE 3:**
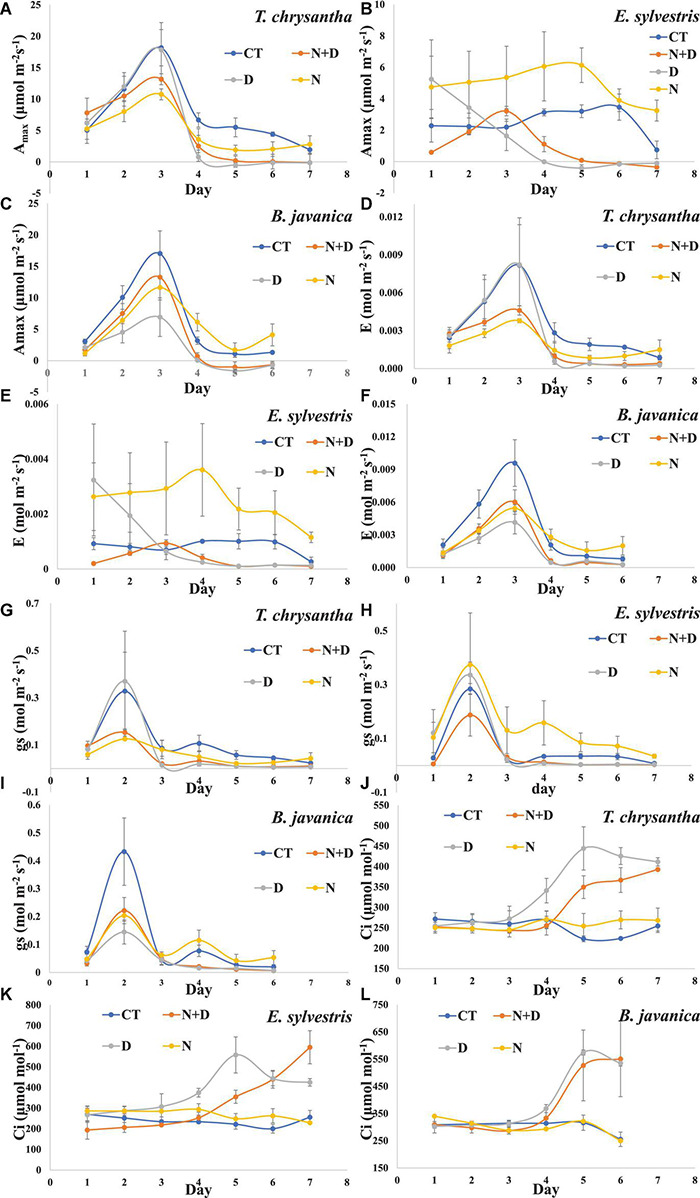
Changes in light-saturated net photosynthetic rate (A_*max*_) **(A–C)**, transpiration rate (E) **(D–F)**, stomatal conductance (g_*s*_) **(G–I)**, intercellular CO_2_ concertation (C_*i*_) **(J–L)** of *Tabebuia chrysantha* **(A,D,G,J)**, *Elaeocarpus sylvestris* **(B,E,H,K)**, and *Bischofia javanica* **(C,F,I,L)** in response to control (CT), nitrogen addition plus drought (N+D), drought (D), and nitrogen addition (N) treatments from day 1 to 7. Error bars were represented standard error (SE).

Drought significantly inhibited the A_*max*_ of the three species, while N addition enhanced the A_*max*_ of the three species, especially for water-sufficient conditions (Figures 3A–C). Except for day 3, the A_*max*_ values of the three plants decreased gradually with the extension of treatment time. This may be related to the environmental conditions on the third day of treatment (such as high humidity, appropriate temperature, and light) more suitable for plant growth. The E and g_*s*_ of the three species decreased significantly under drought conditions. In addition, when the water was sufficient, N supply significantly increased the E and g_*s*_ of *E. sylvestris* and slightly increased that of *B. javanica*, However, N feeds only slightly enhanced the E and g_*s*_ of *B. javanica* under the condition of water shortage (Figures 3D–I). The Ci of the three species increased significantly under drought conditions, while N addition could effectively slow down this increase of *T. chrysantha* and *E. sylvestris* in a certain period of time (Figures 3J–L).

### Instantaneous Carboxylation Efficiency of CO_2_ Assimilation (CE_*i*_), Intrinsic Water-Use Efficiency (WUE_*i*_), and Instantaneous Water-Use Efficiency (WUE_*n*_)

**FIGURE 4:**
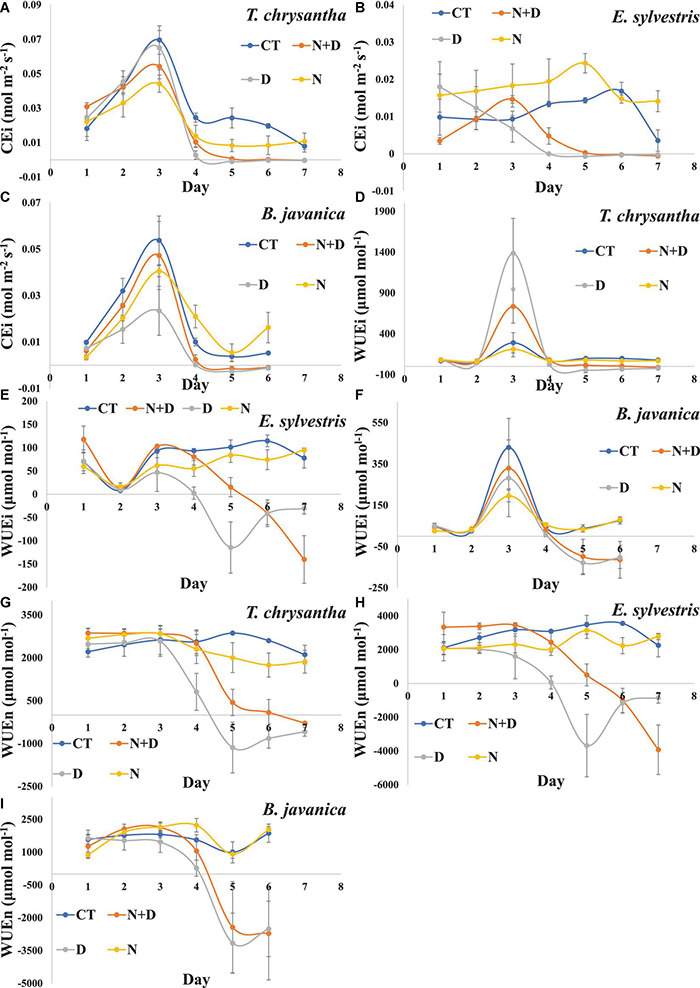
Changes in Instantaneous carboxylation efficiency of CO_2_ assimilation (CE_*i*_) **(A–C)**, intrinsic water-use efficiency (WUE_*i*_) **(D–F)**, instantaneous water-use efficiency (WUE_*n*_) **(G–I)** of *Tabebuia chrysantha* **(A,D,G)**, *Elaeocarpus sylvestris* **(B,E,H)**, and *Bischofia javanica* **(C,F,I)** in response to control (CT), nitrogen addition plus drought (N+D), drought (D), and nitrogen addition (N) treatments from day 1 to 7. Error bars were represented SE.

Drought treatment had a significant inhibition effect on the CEi of the three species. N addition significantly enhanced the CEi of *E. sylvestris* and *B. javanica* under water sufficient conditions, and insignificantly enhanced the CEi of *E. sylvestris* and *B. javanica* while under drought conditions (Figures 4A–C). Compared to CT, the WUEi and WUEn of the three species significantly decreased under drought treatment. In addition, N supply did not significantly affect the WUEi and WUEn of the three species under water-sufficient conditions, but significantly enhanced that of *E. sylvestris* and *B. javanica* under water shortage conditions (Figures 4D–I).

### The Number of Surviving Seedlings

During the experiment, seedling death occurred only in groups D and N+D. As shown in Table 3, on day 5, there was no seedling death in each species group. After day 5, there were two seedlings less in *T. chrysantha* and *E. sylvestris* in the D group. On day 7, only three seedlings of *T. chrysantha* and *E. sylvestris* survived and all seedlings of *B. javanica* died in the D group. Meanwhile, in group N+D, 17 seedlings of *T. chrysantha* survived, 23 seedlings of *E. sylvestris*, and eight seedlings of *B. javanica*. After day 7, all the seedlings of the three species in group D died. In group N+D, there were still 11 seedlings of *T. chrysantha* survived, 16 seedlings of *E. sylvestris* and five seedlings of *B. javanica*.

**TABLE 3:** Number of surviving seedlings of each species in the drought stress (D) group and drought plus nitrogen addition (N+D) group in this experiment (During the experiment, only seedling death occurred in these two groups).

Day	Species	Number of surviving seedlings	
		D	N+D
Day 5	*T. chrysantha*	25	25
	*E. sylvestris*	25	25
	*B. javanica*	25	25
Day 6	*T. chrysantha*	23	25
	*E. sylvestris*	23	25
	*B. javanica*	25	25
Day 7	*T. chrysantha*	3	17
	*E. sylvestris*	3	23
	*B. javanica*	0	8
Day 8	*T. chrysantha*	0	11
	*E. sylvestris*	0	16
	*B. javanica*	0	5

*N+D, drought plus nitrogen addition group; D, drought group.*

## Discussion

### Nitrogen Assimilation and Plant Stress Resistance

Previous studies showed that NH_4_^+^ has strong oxidative toxicity and plants can take up NH_4_^+^ through GS-GOGAT system organically (Li S. et al., 2020). NO_3_^–^ could not be directly utilized by plants, and plants reduce nitrate by NR and NiR for assimilation (Li S. et al., 2020). Cao et al. (2018) and Li S. et al. (2020) found that N supply could enhance the activities of NR, NiR, and GS in plants under D. Consistent with the previous studies, we found that N supply could enhance the activities of NR, NiR, and GS in the three species, mainly because the increased N input promotes the process of N assimilation. As high levels of NH_4_^+^ are toxic to plants and the NH_4_^+^ of the fertilizer would be used by plants several days to weeks later after being transformed to NO_3_^–^. Thus, in this short-term experiment, more effects are from NO_3_^–^ or form. In addition, we also found that drought treatment could significantly increase the activities of these three enzymes in the leaves of the three plants, although the increase was lower than that of the N treatment. N addition greatly enhanced the three enzyme activities of the three species under D. This indicated that plants increase the demand for N assimilation under drought conditions as NO_3_^–^ and NH_4_^+^ are the substrates (Cao et al., 2018). It is possible that the water shortage environment limits the ability of plants to absorb N (Luo and Luo, 2017), thus the activities of these enzymes have not been greatly improved due to the restricted substrate concentration under drought treatment, and the situation is alleviated when N is supplied. The increased requirement for N assimilation during water shortage is closely related to that plants rely on the secretion of amino acids and proteins to resist D (Farooq et al., 2009), while N is the necessary chemical composition of these compounds. Meanwhile, another possible reason for this is that as a high energy-consuming reaction, NO_3_^–^ reduction in leaves can be more efficient and protect the photosynthetic system by using surplus energy in the photosynthetic system under stress (Gonzalez-Dugo et al., 2010).

Drought causes osmotic stress in plants and induces the production of reactive oxygen species (ROS), such as O_2_^–^, H_2_O_2_, and OH^–^, which can destroy cell structure and produce MDA (Munné-Bosch and Penuelas, 2003; Bartels and Sunkar, 2005). Therefore, MDA is an important indicator to measure the damage degree of a plant under oxidative stress (Cao et al., 2014). Saneoka et al. (2004) and Song et al. (2019) found leaf MDA decreased significantly when N was supplied under drought conditions. In this study, we found that the N treatment significantly increased the MDA concentration of the three species, probably due to the oxidative toxicity of NH_4_^+^. The drought treatment increased the MDA concentration of the three species, and when N was added, the MDA concentration significantly decreased under D, indicating that N addition can reduce the destruction of cell structure thus enhancing the tolerance of plants to D in subtropical evergreen broad-leaved forests. Guo et al. (2010) and Iqbal et al. (2020) also found that N addition can reduce the MDA concentration of the plants to enhance the tolerance of the plants to D in tropical and temperate zones. In this experiment, N application effectively reduced the mortality of the three plant species under drought treatment, and N application can improve the tolerance of plants to D on a certain time scale.

In response to oxidative stress, plants secrete antioxidants to restore the homeostasis between the production and scavenging of ROS, so as to reduce the damage (Cao et al., 2014). Previous studies (Yao and Liu, 2006; Ochoa-Velasco et al., 2016) found that N application could apparently enhance the antioxidant enzyme activity of plants. We also found that the leaf T-AOC under N treatment was significantly higher than that of CT in the three tree species. In contrast, the N addition obviously decreased the T-AOC in each species under drought conditions in this experiment, which was in accordance with the results in the research of Song et al. (2019). The MDA concentration and T-AOC were significantly negatively correlated with PRO concentration in each species under both drought and N addition plus drought treatments in the current study. These results manifested that the reduction of MDA concentration of the three species was not mainly due to the enhancement of antioxidant activity when N was added under D. It is possible that N addition alleviated osmotic stress and effectively protected the structure of cells (Ashraf and Foolad, 2007; Cao et al., 2014), such that the oxidative damage and antioxidants were all reduced subsequently (Ashraf and Foolad, 2007; Farooq et al., 2009).

Plants increase the production of osmolytes (such as glycine betaine, PRO, and soluble sugars) to reduce osmotic potential and protect the structure of cells in response to osmotic stress in dry environments (Ashraf and Foolad, 2007; Foyer and Shigeoka, 2011). Song et al. (2019) found that PRO concentration was dramatically enhanced by N addition in plants under dehydrated conditions. It was also found in temperate zones by the greenhouse and field experiments that the increase of PRO concentration can be induced by N fertilization to enhance the tolerance of the plants to D (Monreal et al., 2007; Albert et al., 2012). Similarly, our results showed that N treatment significantly increases the PRO concentration of the three tested tree species. Drought treatment also significantly increased the PRO content in plants, and N addition can promote the synthesis of PRO though under drought conditions. The most probable mechanism is that N addition increases nitric oxide (NO) concentration in the plants through the N assimilation pathway (Chen et al., 2012), then NO induces the increase of PRO at the transcription level (Zhao et al., 2009; Puyaubert and Baudouin, 2014). In this study, the content of PRO also showed a strong positive correlation with NR and NiR. This study showed that in a subtropical evergreen broad-leaved forest, inducing the increase of plant proline is an important process for N addition to improving the tolerance of plants to D.

From our results, in dealing with oxidative stress caused by osmotic stress, the three species seemed to prefer osmotic regulation rather than enhancing antioxidant capacity in subtropical evergreen broad-leaved forests. Guo et al. (2010) and Li S. et al. (2020) also found that N addition reduced MDA concentration and antioxidant capacity of the plants under D in tropical and subtropical zones. However, N addition enhanced antioxidant capacity and reduced MDA concentration of the plants under D from the experiment of Iqbal et al. (2020) in temperate zones. Therefore, there might be different trade-off mechanisms (osmotic adjustment vs. antioxidant capacity) for tropical and subtropical plants and temperate plants to deal with the destruction of cell structure caused by D and N supply (Greaver et al., 2016; Lu et al., 2021).

### Leaf Gas Exchange Performances

Many studies have shown that N supply can increase the net photosynthesis rate of plants (Liu et al., 2011; Luo and Luo, 2017; Schulte-Uebbing and de Vries, 2018; Liang et al., 2020). In plants, N is majorly stored in enzymes that are involved in photosynthesis, especially Rubisco, the key link of carbon assimilation and the N cycle (Abid et al., 2016). The increase of Rubisco content and activity is also an important part of the mechanism of N addition to increasing the net photosynthetic rate of plants (Abid et al., 2016). In this study, N application also increased the Rubisco activity of the three species and significantly enhanced the A_*max*_ of *E. sylvestris* and *B. javanica*. This manifests that N addition could strengthen the capacity of C assimilation of the two species, and may further alter the C cycle of the ecosystem in subtropical evergreen broad-leaved forests. This is consistent with previous findings that N deposition can increase carbon sequestration in ecosystems by reducing the efflux of CO_2_ and dissolved organic carbon and increasing net primary productivity and litter (Lu et al., 2021). Under D, the carboxylation efficiency of Rubisco is greatly reduced, acting more as an oxygenase than a carboxylase, which reduces photosynthetic carbon assimilation but protected the photosynthetic system from the damage of residual light energy (Makino, 2011). Pankovic et al. (1999) and Demirevska et al. (2008) have found that the content of Rubisco in plants, especially drought-tolerant species, increases under water deficit conditions, which was thought to probably enhance the photorespiration of the plants. In this study, under the condition of water shortage, the enhancement of Rubisco enzyme activities by N probably also be an important process for N to enhance plant tolerance to drought.

The stomata is another important factor, which regulates the inlet and outlet of gas in leaves (Albert et al., 2012). For photosynthesis, when the substrate CO_2_ of Rubisco is insufficient, larger g_*s*_ is needed to absorb more CO_2_ to improve the reaction, or g_*s*_ would become a limiting factor for photosynthesis (Zhong et al., 2019). Under the condition of water deficit, non-stomatal limitation plays a major role in photosynthesis (Gallé et al., 2007; Singh and Raja Reddy, 2011; Mashilo et al., 2018). In this study, the Ci of drought treatment was higher than that of the control group, which also supported this finding. The A_*max*_ of the plants in the N plus drought group were lower than that in N and control groups, however, the Ci of the plants did not show the same trend, or even the opposite, indicating that non-stomatal limitation is still the dominant limiting factor of photosynthesis in an arid environment even under the fertilization of N (Mashilo et al., 2018).

Transpiration (E) is an indicator affected by g_*s*_, which operates through stomata (Liang et al., 2020). Lu et al. (2018) found that N deposition would reduce the availability of basic cations, such as calcium ions (Ca^2+^) and magnesium ions (Mg^2+^), in soil, reduce stomatal closure, increase transpiration (E), and enhance the capacity of plants to uptake water and soluble substances in the soil in N-rich forest ecosystem. In this experiment, E basically changed synchronously with g_*s*_. N addition significantly enhanced the E of *E. sylvestris* under water-sufficient conditions, but only slightly increased the E of *T. chrysantha* and *B. javanica* under water-insufficient conditions. It shows that N addition can promote plant transpiration under drought conditions. However, the impact level of different species is discrepant and this is probably caused by the different sensitivity of different species to water shortage (Shi et al., 2017; Lu et al., 2018; Song et al., 2019).

In this experiment, the WUEi and WUEn of plants were calculated by the ratio of A_*max*_ to g_*s*_ and to E, respectively, which represented different water use impact models (Field et al., 1982, 1983). The instantaneous water use efficiency was found not to be significantly altered by N addition at the global level in the meta-analysis of Liang et al. (2020). Perhaps because A_*max*_, E, and g_*s*_ were all elevated under N application (Shi et al., 2017). N addition also did not significantly change the WUEi and WUEn of the three species in this research. Although A_*max*_, g_*s*_, and E of the three species were all inhibited under drought conditions, the WUEn and WUEi in drought treatment were significantly lower than that in the control group. This indicated that in addition to stomatal limitation, photosynthesis was also restricted by many factors, such as oxidative stress (Saud et al., 2016). Under drought conditions, N supply only increased the WUE of *E. sylvestris*, because N increased its photosynthesis but did not change its g_*s*_ and E, showing that N addition can maintain the growth of *T. chrysantha* and *B. javanica* by increasing g_*s*_ and E, and the growth of *E. sylvestris* was maintained by increasing the proportion of water consumed in photosystem (Shi et al., 2017; Yu-Zheng et al., 2021).

Through the depolarization of guard cells, NO_3_^–^ has been testified to be involved in regulating stomatal opening (Raddatz et al., 2020), and ABA is a key factor regulating stomatal closure (GarcıìA-Mata and Lamattina, 2002). ABA is commonly considered to increase in leaves under D to regulate stomatal closure to reduce plant transpiration and water loss, and also to participate in the regulation of antioxidant enzymes (Cao et al., 2012; Gallé et al., 2013; De Vries et al., 2016). Previous studies have shown that N deposition promotes stomatal opening to obtain carbon dioxide (Lu et al., 2018; Liang et al., 2020). We also found that drought significantly elevated the level of ABA and significantly decreased stomatal conductance. The N application was found to significantly increase the ABA of *E. sylvestris* and *B. javanica* under drought, however, only slightly affect the g_*s*_ of *B. javanica* on day 2, while N addition insignificantly increased the g_*s*_ of *T. chrysantha* and *B. javanica* under water-sufficient conditions by the end of the trial. These results show that when water was deficient, the effect of N addition on the g_*s*_ of the plants might be weakened (Albert et al., 2012; Shi et al., 2017). The function of ABA regulating stomatal closure needs to be completed together with NO (GarcıìA-Mata and Lamattina, 2002; Neill, 2002), which is majorly produced by the NR-NiR pathway (Desikan et al., 2002). Therefore, we suggest that plants may use the NR-NIR pathway to control their NO-NO_3_^–^ balance, and cooperate with ABA and other regulatory factors to regulate stomatal opening and closing to respond to internal demand and external environment while under N addition.

Nitrogen deposition is a long-term process. The response and acclimation of subtropical evergreen broad-leaved forest tree species in South China to the increasing N deposition also experienced a long period (Bobbink et al., 2010). Thus the 7-day experimental period in this study is too short to fully reflect the response of subtropical evergreen broad-leaved forest tree species in south China to continuously enhanced N deposition. With this limitation, however, the current study still has a certain indicative effect on the response of subtropical evergreen broad-leaved forests to N deposition in south China as the seedling establishment is the key stage for plants to acclimate to the environment (Grossnickle and MacDonald, 2018). In this experiment, N deposition promotes plant photosynthesis capacity (A_*max*_, Rubisco activity), osmotic regulation (PRO), and avoidance of excess light energy (Rubisco, transpiration), and thus reduces the MDA, improving the drought tolerance of the three plants of the three tree species under drought conditions. This helps reflect the internal mechanism of the “fertilization effect” of evergreen broad-leaved forest in south China in the short-term stage of N deposition (Wortman et al., 2012).

## Conclusion

In this study, N addition can improve the drought tolerance of the tree species to some extent by alleviating osmotic stress and protecting photosynthetic apparatus under drought conditions in short term, and the mechanism is to promote the production of osmolytes, such as PRO, and induce the nitrate reduction and photorespiration in leaves. N addition promoted photosynthesis, transpiration, and the stomatal opening of the plants under D from the beginning to the middle of the experiment. Later in the experiment, however, this promotion decreased or even disappeared. The elevation of photosynthesis and transpiration means more consumption and loss of water, which is not conducive to the acclimation of plants to a drought environment and may even aggravate the water shortage in the environment and within plants under mid-term N deposition.

We also found that *B. javanica* (large tree) has a higher improvement in photosynthesis and transpiration under D. This means that the elevation of water consumption by N addition to *B. javanica* was higher than that of the other two species, and may help explain why the seedlings of *B. javanica* died one day earlier than the other two species. Furthermore, these results can also help to explain the degradation and why large tree species of evergreen broad-leaved forest are difficult to adapt to climate change. This study also helps in explaining the findings in previous research that the tropical and subtropical evergreen broad-leaved forests in South China are gradually degraded into shrublands (Zhou et al., 2014). On a long-term scale, N deposition may lead to water deficiency in plants and soil water shortage and eventually lead to the degradation of evergreen broad-leaved forests in South China.

## Data Availability Statement

The original contributions presented in the study are included in the article/supplementary material, further inquiries can be directed to the corresponding author.

## Author Contributions

FL and SZ collected and analyzed the data. FL, YZ, and NL led the writing of the manuscript. All authors conceived the ideas, designed methodology, contributed critically to the drafts, and gave final approval for publication.

## Conflict of Interest

The authors declare that the research was conducted in the absence of any commercial or financial relationships that could be construed as a potential conflict of interest.

## Publisher’s Note

All claims expressed in this article are solely those of the authors and do not necessarily represent those of their affiliated organizations, or those of the publisher, the editors and the reviewers. Any product that may be evaluated in this article, or claim that may be made by its manufacturer, is not guaranteed or endorsed by the publisher.

## Footnotes

1
https://www.r-project.org/

2
https://www.ibm.com/cn-zh/analytics/spss-statistics-software
